# Recent Advances in Biological and Technological Research of Fresh Fruit and Vegetable: Third Edition

**DOI:** 10.3390/foods15213966

**Published:** 2026-11-05

**Authors:** Zhongqi Fan

**Affiliations:** College of Food Science, Fujian Agriculture and Forestry University Fujian, Fuzhou 350002, China; fanzqi@fafu.edu.cn

Fresh fruit and vegetables remain metabolically active after harvest and are susceptible to chilling injury, senescence, and microbial decay, all of which can reduce quality and shorten their marketable life [1,2]. Building on the previous two editions of this Special Issue [3,4], the third edition brings together seven original research articles concerning fruit, leafy vegetables, and edible fungi. The contributions address molecular regulation, tissue and membrane changes, antioxidant responses, disease resistance, and preservation treatments. Together, they connect the biology of postharvest deterioration with ways to maintain the quality of perishable foods.

Two contributions examine mechanisms underlying chilling injury and textural deterioration. Liang et al. [contribution 1] found that melatonin treatment maintained higher expression of *EjMYB15* in cold-stored loquat fruit. EjMYB15 bound to and activated the promoters of four antioxidant enzyme genes, supporting a role for antioxidant gene regulation in alleviating chilling injury. In shiitake mushrooms, Gao et al. [contribution 2] combined physiological measurements with transcriptome analysis and associated textural deterioration with changes in cell wall components, hydrolytic enzyme activities, and reactive oxygen species balance. Their analysis implicated laccase-related genes in a potential regulatory network for cell wall remodeling, although functional experiments are still needed to establish their roles. These studies identify molecular processes that may inform future approaches to quality maintenance.

Several studies evaluate exogenous compounds against different forms of quality loss. Shi et al. [contribution 3] found that glycine betaine treatment maintained firmness and reduced peel reddening in cold-stored ‘Hupingzao’ jujubes, alongside lower oxidative damage and greater antioxidant capacity. In button mushrooms, Xu et al. [contribution 4] reported that arginine delayed softening, with changes in lipid metabolism and membrane integrity associated with this effect. Chen et al. [contribution 5] showed that thyme essential oil delayed leaf yellowing and chlorophyll loss in Chinese flowering cabbage while helping to retain vitamin C and other quality attributes. The varied responses underline the need to select treatments according to the deterioration processes and quality requirements of each commodity.

Physical treatment offers another route to preserving quality. Chen et al. [contribution 6] evaluated electron beam irradiation of passion fruit and found that 0.2 kGy was the most effective of the tested doses. Treated fruit showed less weight loss and lower relative electrical conductivity, with a higher proportion remaining commercially acceptable during storage. Irradiation also limited superoxide production and membrane lipid peroxidation, slowed the loss of phospholipids and unsaturated fatty acids, and enhanced antioxidant capacity. These results associate improved storage quality with better maintenance of membrane lipid composition and redox balance; the dose-specific response also warrants attention when adapting the treatment to other commodities.

Postharvest disease resistance is a further concern. Inducing the defenses of harvested fruit is an established area of research for limiting pathogen development [5]. Zhu et al. [contribution 7] found that ethephon treatment reduced disease severity and pericarp browning in litchi fruit inoculated with *Peronophythora litchii*. Treated fruit produced more ethylene, showed higher activity of antioxidant and defense-related enzymes, and accumulated less hydrogen peroxide and malondialdehyde. These observations are consistent with stronger host defense and reduced oxidative damage contributing to disease suppression, while further work is needed to assess the treatment under commercial conditions.

Across the seven contributions, antioxidant balance, membrane integrity, and tissue structure recur as factors associated with postharvest quality; the litchi study additionally highlights host responses to infection. The intervention studies show that exogenous compounds and physical treatment can address distinct forms of deterioration. Priorities for future work include functional validation of proposed regulatory networks, optimization of treatment conditions, and evaluation during commercial storage and distribution. By linking biological responses to practical preservation approaches, this Special Issue provides a basis for more targeted strategies for fruit, vegetables, and edible fungi. I thank the contributing authors, reviewers, and the *Foods* Editorial Office for their efforts in bringing this Special Issue together.
